# Metabolic Syndrome, Neurotoxic 1-Deoxysphingolipids and Nervous Tissue Inflammation in Chronic Idiopathic Axonal Polyneuropathy (CIAP)

**DOI:** 10.1371/journal.pone.0170583

**Published:** 2017-01-23

**Authors:** Larissa Hube, Maike F. Dohrn, Gergely Karsai, Sarah Hirshman, Philip Van Damme, Jörg B. Schulz, Joachim Weis, Thorsten Hornemann, Kristl G. Claeys

**Affiliations:** 1 Department of Neurology, RWTH Aachen University, Aachen, Germany; 2 Institute of Neuropathology, RWTH Aachen University, Aachen, Germany; 3 Center for Integrative Human Physiology, University of Zurich, Zurich, Switzerland; 4 Institute for Clinical Chemistry, University Hospital, Zurich, Switzerland; 5 Department of Neurology, University Hospitals Leuven, Leuven, Belgium; 6 Department of Neurosciences, Experimental Neurology, University of Leuven (KU Leuven), Leuven, Belgium; 7 VIB, Vesalius Research Center, Laboratory of Neurobiology, Leuven, Belgium; 8 JARA-Institute Molecular Neuroscience and Neuroimaging, Forschungszentrum Jülich GmbH and RWTH Aachen University, Aachen, Germany; 9 Department of Neurosciences, Experimental Neurology, Laboratory for Muscle Diseases and Neuropathies, University of Leuven (KU Leuven), Leuven, Belgium; Szegedi Tudomanyegyetem, HUNGARY

## Abstract

**Aim:**

Chronic idiopathic axonal polyneuropathy (CIAP) is a slowly progressive, predominantly sensory, axonal polyneuropathy, with no aetiology being identified despite extensive investigations. We studied the potential role of the metabolic syndrome, neurotoxic 1-deoxysphingolipids (1-deoxySLs), microangiopathy and inflammation in sural nerve biopsies.

**Methods:**

We included 30 CIAP-patients, 28 with diabetic distal symmetrical polyneuropathy (DSPN) and 31 healthy controls. We assessed standardised scales, tested for the metabolic syndrome, measured 1-deoxySLs in plasma, performed electroneurography and studied 17 sural nerve biopsies (10 CIAP; 7 DSPN).

**Results:**

One third of the CIAP-patients had a metabolic syndrome, significantly less frequent than DSPN-patients (89%). Although the metabolic syndrome was not significantly more prevalent in CIAP compared to healthy controls, hypercholesterolemia did occur significantly more frequent. 1-deoxySLs were significantly and equally elevated in both patient groups compared to healthy controls. Mean basal lamina thickness of small endoneurial vessels and the number of CD68- or CD8-positive cells in biopsies of CIAP- and DSPN-patients did not differ significantly. However, the number of leucocyte-common-antigen positive cells was significantly increased in CIAP.

**Conclusions:**

A non-significant trend towards a higher occurrence of the metabolic syndrome in CIAP-patients compared to healthy controls was found. 1-deoxySLs were significantly increased in plasma of CIAP-patients. Microangiopathy and an inflammatory component were present in CIAP-biopsies.

## Introduction

Chronic idiopathic axonal polyneuropathy (CIAP) is defined as a slowly progressive, predominantly sensory polyneuropathy with an axonal pattern in nerve conduction studies (NCS), in which all known causes were excluded [1]. Recent studies suggested that disorders in lipid and glucose metabolism, known as the metabolic syndrome [2], are more prevalent in CIAP-patients [3–6]. Further arguing for a metabolic source is the clinical similarity of CIAP to diabetic distal symmetrical polyneuropathy (DSPN) [3]. Moreover, impaired glucose tolerance (IGT) or prediabetes recently became a recognised cause of axonal polyneuropathy, which underlines the relevance of metabolic factors in the development of neuropathies [7–9].

A recent study links DSPN with elevated 1-deoxysphingolipids (1-deoxySLs) in blood [10]. Sphingolipids play an important role in the formation of plasma membranes and lipoproteins; they are usually formed by the precursors L-serine and palmitoyl-CoA. Their condensation is catalysed by the enzyme serine palmitoyltransferase (SPT) leading to the formation of sphinganine. However, SPT is also able to metabolise L-alanine and L-glycine generating neurotoxic 1-deoxySLs. These metabolites can neither be used to form complex sphingolipids nor can they be degraded [11, 12]. The neurotoxic effect of 1-deoxySLs was demonstrated on cultured sensory neurons [12]. Considering the similarities with DSPN, the presence of 1-deoxySLs might also play a role in the pathomechanism of CIAP.

To date, knowledge on the histopathological characteristics in CIAP is limited. Increased basal lamina thickness of endothelial cells in CIAP resembling the microangiopathy observed in nerve biopsies of DSPN-patients has been reported [13, 14]. However, biopsy data on other possible disease factors, such as inflammation, are lacking.

We studied the potential role of the metabolic syndrome, elevated plasma 1-deoxySLs and the occurrence of microangiopathy and inflammatory changes in sural nerve biopsies in a cohort of 30 CIAP-patients compared to DSPN-patients and healthy controls.

## Patients and Methods

### Patients and controls

We included 30 CIAP-patients followed at our neuromuscular clinic (RWTH University Hospital Aachen, Germany). Diagnosis of CIAP was based upon the following criteria: 1) primarily sensory, symmetrical neuropathy of the distal limbs with no signs of weakness except for mild toe and/or finger weakness; 2) dysesthesia, loss of sensation, temperature or vibration sense; 3) hypo- or areflexia may be present, as well as symptoms of gait unsteadiness and autonomic dysfunction; 4) symptoms for >3 months; 5) no demyelinating abnormalities at NCS; 6) no other identifiable cause, including metabolic, toxic, infectious, systemic (e.g. vasculitis) or hereditary causes; or in case of a monoclonal gammopathy, a lymphoproliferative disorder, malignancy or amyloidosis [1].

Two age-and-sex-matched control groups were included. The first comprised 28 patients with diabetic polyneuropathy due to type 2 diabetes mellitus (DM2; n = 19) or prediabetic polyneuropathy (n = 9). Prediabetes or IGT was defined as: 1) plasma glucose on 2-h-oral-glucose-tolerance test (oGTT) between ≥140 and <200 mg/dl, and 2) fasting glucose >126 mg/dl [7, 15]. The second control group included 31 healthy individuals.

Written informed consent was obtained. The study was performed according to the Declaration of Helsinki and approved by the ethical committee of the RWTH University Hospital Aachen.

### Clinical examination and nerve conduction studies (NCS) in CIAP-patients

A detailed history was obtained and neurological and clinical examination, including blood pressure and waist circumference, were performed. Tibial anterior, gastrocnemius, peroneal and toe extensors muscle strength were manually tested bilaterally using the Medical Research Council (MRC) scale (motor sum score 0–40; total paralysis-normal strength) [3]. For quantification of sensory symptoms, we applied the Inflammatory Neuropathy Cause and Treatment Sensory Sum Score (ISS; 0–20; no-most severe sensory deficit). The score comprises measurement of pinprick and vibration sensation in arms and legs on both sides using the Rydel-Seiffer graduated tuning fork and bilateral two-point discrimination on the ventral side of the index finger [16]. A mobility scale (0–5; normal-total immobility) [17], and the Overall Neuropathy Limitations Scale (ONLS) to quantify physical impairment (0–12; no-most severe disability) [18] were applied. A visual pain scale (0–10; no-worst neuropathic pain), Fatigue Severity Scale (FSS) [19] and Beck-Depressions-Inventar-II (BDI-II; >9 = possible depression) [20] were performed. NCS of both tibial and sural nerves were conducted in all patients.

### Blood analyses in CIAP-patients

Blood analysis was performed in patients to exclude underlying causes of neuropathy [21]. Laboratory studies included a blood count, C-reactive protein, glucose, glycated haemoglobin (HbA_1c_), renal and liver function, sodium, potassium, calcium, TSH, fT4, carbohydrate-deficient transferrin (CDT), serum protein electrophoresis and immune fixation, ANA, ANCA, rheumatoid factor, cyclic citrullinated peptides-antibody, angiotensin-converting enzyme, soluble IL-2 receptor, hepatitis B and C, folic acid, vitamin B12, methylmalonic acid, transcobolamin, total cholesterol, HDL- and LDL-cholesterol, triglyceride levels and uric acid.

An oGTT was performed in CIAP-patients to rule out DM2 or IGT [7].

The metabolic syndrome was defined by at least three of the following criteria: 1) waist circumference ≥102 cm in men, ≥88 cm in women; 2) triglycerides (TG) ≥150 mg/dl in blood or lipid-lowering drug treatment; 3) HDL-cholesterol ≤40 mg/dl in men, ≤50 mg/dl in women or lipid-lowering drug treatment; 4) systolic blood pressure ≥130 mmHg or diastolic blood pressure ≥85 mmHg or antihypertensive medication; 5) fasting glucose ≥100 mg/dl or drug treatment for DM2 [2].

### Analysis of plasma sphingolipids in CIAP-patients and controls

The following sphingoid bases were analysed in hydrolysed plasma in all patients and controls as previously described [10, 11]: C_16_SO, C_16_SA, C_17_SO, C_17_SA, C_19_SO, C_20_SO, C_20_SA, phytoSO, sphingadiene and the two 1-deoxySLs: 1-deoxysphingosine (1-deoxySO) and 1-deoxysphinganine (1-deoxySA). Cut-off values for 1-deoxySO and 1-deoxySA were determined using lipid profile data from a large cohort of healthy individuals (n = 437), that were analysed under identical standards in the same laboratory (Institute for Clinical Chemistry, University Hospital Zurich, Switzerland). Values >90^th^ percentile of this reference group were considered to be abnormal. The cut-off was set at 0.26 μmol/l for 1-deoxySO and 0.13 μmol/l for 1-deoxySA.

### Examinations in control groups

The 28 control patients with (pre-)diabetic polyneuropathy underwent the same clinical exams, scores and NCS as CIAP-patients. In 31 healthy controls, history was taken and measurement of blood pressure and waist circumference performed. In both control groups, total cholesterol, HDL-, LDL-cholesterol, TG-levels and sphingolipids were analysed.

### Microscopic nerve biopsy study

We studied 10 sural nerve biopsies from CIAP-patients and seven from DSPN-patients (DM2 n = 4; prediabetic n = 3), that were obtained for diagnostic purposes prior to the study, and processed for light and electron microscopy following standard procedures [22]. Immunohistochemical stains with antibodies against CD8, CD68 and leucocyte common antigen (LCA) were performed.

To evaluate the small endoneurial vessels and the presence of microangiopathy, we investigated semithin sections (0.5 μm), which were stained with 1% toluidine blue, at 63-fold magnification using an Axio Scope.A1 (Carl Zeiss Microscopy GmbH, Jena, Germany), and counted the number of LCA-, CD8- and CD68-positive cells. The complete section through the biopsy was evaluated. Furthermore, the number of endothelial cell nuclei adhering to the lumen or located within the basement membrane, as well as the thickness of the basement membrane of each small endoneurial vessel [23], was evaluated by viewing the specimens in semithin sections. The results of this quantitative analysis of inflammatory cells, endothelial cell nuclei and basement membrane thickness were correlated to the number of fascicles in each section to obtain mean results per fascicle. The small endoneurial vessels were also pictured using ultra-thin sections (60 nm) and electron microscopy (Philips CM10, Philips Deutschland, Hamburg, Germany). The vessels were photographed, dependent on their size, at magnifications varying between 3,400 and 13,500.

### Statistical analysis

Statistical analysis was conducted using GraphPad Prism Version 6. To test for normal distribution, we used Kolmogorow-Smirnow, D’Agostino and Pearson omnibus and Shapiro-Wilk normality tests. If data were normally distributed, groups were compared by one-way ANOVA (using Tukey-Kramer post-test method to correct p-levels) or unpaired t-test. When data were not normally distributed, log transformation was performed and normal distribution was reassessed. If still not confirmed, we applied the Kruskal-Wallis test or Mann-Whitney U test instead. Categorical variables were compared using Fisher’s exact test. Statistical significance was set at P ≤ 0.05.

Regression analysis using non-linear regression was performed comparing HDL-, LDL-cholesterol, triglyceride-levels and 1-deoxySLs with clinical and paraclinical parameters. Regression analysis was also done for basement membrane thickness in comparison with the levels of 1-deoxySLs, cholesterol, triglycerides and blood pressure.

## Results

### Demographics in CIAP and controls (Table 1)

**Table 1:** Demographic and clinical features in CIAP patients and controls.

Parameter	CIAP	DSPN	Healthy Controls	P
Number	30	28	31	ns
Age (y)	61.1 ± 9.5	61.0 ± 9.3	60.0 ± 11.3	ns
F/M (%)	14/16 (47/53)	11/17 (39/61)	14/17 (45/55)	ns
Duration of symptoms (years)	8.5 ± 5.4	5.6 ± 3.9	/	0.03 (*)
ISS	4.0 ± 2.2	4.3 ± 2.1	/	0.7 (ns)
ONLS	1.1 ± 1.2	1.9 ± 2.0	/	0.06 (ns)
Mobility test (s)	1.1 ± 0.3	1.6 ± 1.2	/	0.053 (ns)
MRC sum score	38.4 ± 2.8	37.5 ± 5.7	/	0.8 (ns)
Visual pain scale	3.5 ± 3.4	4.4 ± 4.3	/	0.003 (**)
Fatigue severity scale	3.6 ± 2.0	4.2 ± 2.2	/	0.4 (ns)
Beck-depression-inventar II	8.8 ± 7.9	12.0 ± 8.3	/	0.07 (ns)
SNAP sural nerve left (μV)	3.9 ± 3.7	4.3 ± 4.8	/	0.9 (ns)
NCV sural nerve left (m/s)	34.0 ± 20.7	32.8 ± 20.7	/	0.8 (ns)
SNAP sural nerve right (μV)	3.9 ± 3.5	4.3 ± 3.4	/	0.7 (ns)
NCV sural nerve right (m/s)	32.3 ± 21.1	38.3 ± 16.8	/	0.9 (ns)

**CIAP**, chronic idiopathic axonal polyneuropathy; **DSPN**, (pre-)diabetic distal symmetrical polyneuropathy; **F**, females; **M**, males; **ISS**, Inflammatory Neuropathy Cause and Treatment Sensory Sum Score; **ONLS**, Overall Neuropathy Limitations Scale; **MRC sum score**, Medical Research Council sum score for muscle strength; **SNAP**, sensory nerve action potential; **NCV**, nerve conduction velocity. Groups of three were compared by one-way ANOVA (using Tukey-Kramer post-test method to correct p-levels), groups of two by unpaired t-test.

The level of statistical significance is indicated as: not significant (ns): P > 0.05; significant:

*: P ≤ 0.05;

**: P ≤ 0.01.

Thirty CIAP-patients (14 females (46.7%) and 16 males (53.3%)) were included with mean age at examination of 61.1 years (±9.5). In our first control group, 19 patients presented with DSPN and nine had a polyneuropathy and IGT. Recent studies have shown that similar to DM2, IGT can lead to a sensory-predominant polyneuropathy [8, 9, 21]. We did not find any significant differences between patients with polyneuropathy due to DM2 or IGT and therefore combined them into the DSPN control group (n = 28). There were no significant differences between number of patients, age and gender distribution between CIAP-, DSPN-patients and healthy controls. CIAP-patients had a significantly longer duration of symptoms than patients with DSPN (Table 1).

### Clinical and NCS findings in CIAP and DSPN (Table 1)

Although 17 CIAP- (60%) and 15 DSPN-patients (53%) complained of neuropathic pain, the visual pain scale revealed significantly less severe pain in CIAP-patients (P = 0.003). There were no significant differences between CIAP- and DSPN-patients concerning (mild) sensory symptoms (ISS), motor function (MRC sum score) or fatigue syndrome (FSS) (Table 1). Comparison of the depression score (BDI II), mobility test and physical impairment (ONLS), revealed a trend towards worse scoring in DSPN-patients compared to CIAP-patients. The sural nerve SNAP and NCV bilaterally were compatible with a sensory polyneuropathy in both groups, but did not show significant differences between CIAP- and DSPN-patients (Table 1).

### Prevalence of the metabolic syndrome in CIAP and control groups (Table 2)

**Table 2:** Prevalence of metabolic syndrome and its components in CIAP patients and controls.

Parameter	CIAP	DSPN	Healthy Controls	Comparison	P	OR
HDL (mg/dl)	63.6 ± 22.3	46.5 ± 15.8	64.6 ± 16.9	CIAP vs. healthy C.	ns	/
LDL (mg/dl)	147.3 ± 31.0	131.7 ± 43.6	125.8 ± 28.1	CIAP vs. healthy C.	*	/
Triglycerides (mg/dl)	152.7 ± 126.2	241.3 ± 144.2	129.2 ± 55.4	CIAP vs. healthy C.	ns	/
BP systolic (mmHg)	127.3 ± 15.6	136.1 ± 19.4	124.0 ± 17.6	CIAP vs. healthy C.	ns	/
BP diastolic (mmHg)	78.2 ± 11.1	80.2 ± 13.2	77.9 ± 11.7	CIAP vs. healthy C.	ns	/
Waist circumference (cm)	103.4 ± 13.6	121.7 ± 16.6	96.2 ± 10.8	CIAP vs. healthy C.	ns	/
Metabolic Syndrome (%)	10 (33.0)	25 (89.2)	6 (19.4)	CIAP vs. healthy C.	0.3 (ns)	2.1
Metabolic Syndrome (%)	10 (33.0)	25 (89.2)	6 (19.4)	CIAP vs. DSPN	< 0.0001 (****)	0.06
Metabolic Syndrome (%)	10 (33.0)	25 (89.2)	6 (19.4)	DSPN vs. healthy C.	< 0.0001 (****)	34.7
Hypercholesterolemia (%)	19 (61.3)	18 (64.3)	6 (19.4)	CIAP vs. healthy C.	0.0007 (***)	7.2
Hypercholesterolemia (%)	19 (61.3)	18 (64.3)	6 (19.4)	CIAP vs. DSPN	1 (ns)	0.96
Hypercholesterolemia (%)	19 (61.3)	18 (64.3)	6 (19.4)	DSPN vs. healthy C.	0.0006 (***)	7.5
Arterial hypertension (%)	18 (61.3)	25 (89.3)	11 (35.5)	CIAP vs. healthy C.	0.07 (ns)	2.7
Arterial hypertension (%)	18 (61.3)	25 (89.3)	11 (35.5)	CIAP vs. DSPN	0.02 (*)	0.18
Arterial hypertension (%)	18 (61.3)	25 (89.3)	11 (35.5)	DSPN vs. healthy C.	< 0.0001 (****)	15.2

**CIAP**, chronic idiopathic axonal polyneuropathy; **DSPN**, (pre-)diabetic distal symmetrical polyneuropathy; **OR**, odds ratio; **HDL**, high-density lipoprotein; **healthy C**., healthy controls; **LDL**, low-density lipoprotein; **BP systolic**, systolic blood pressure; **BP diastolic**, diastolic blood pressure. Definition of metabolic syndrome is based on the Adult Treatment Panel II criteria: 1) elevated waist circumference 2) elevated triglycerides 3) reduced HDL-cholesterol 4) elevated systolic blood pressure 5) elevated fasting glucose (see text). Numerical values were compared by one-way ANOVA (using Tukey-Kramer post-test method to correct p-levels). Categorical v0061riables, such as prevalence of metabolic syndrome, were compared using Fisher’s exact test. Level of statistical significance is indicated as: not significant (ns): P > 0.05; significant:

*: P ≤ 0.05;

**: P ≤ 0.01;

***: P ≤ 0.001;

****: P ≤ 0.0001.

The criteria of the metabolic syndrome were fulfilled in 10 CIAP-patients (33%), which is significantly less than in 25 DSPN-patients (89.2%) (P<0.0001; Odds ratio (OR) = 0.06) (Table 2). The metabolic syndrome was not significantly more prevalent in CIAP-patients compared to healthy controls (n = 6, 19.4%; P = 0.3; OR = 2.1).

The assessment of the individual components of the metabolic syndrome criteria, however, showed a significantly higher prevalence for hypercholesterolemia (i.e. raised LDL-cholesterol ≥150 mg/dl, reduced HDL-cholesterol ≤40 mg/dl in men or ≤50 mg/dl in women, and/or usage of lipid lowering agents) in CIAP-patients compared to healthy controls (P = 0.0007; OR = 7.2) (Table 2).

In CIAP-patients, neither arterial hypertension was significantly more prevalent, nor did we find significantly raised plasma TG-levels or a larger waist circumference compared to healthy participants, although there was a trend towards higher values (Table 2).

### Analysis of sphingolipids in CIAP and control groups (Table 3, Fig 1)

**Table 3:** Plasma sphingolipid profile in CIAP patients and controls.

Parameter	CIAP	DSPN	Healthy Controls	CIAP vs. DSPN	CIAP vs. healthy Controls
C16SO (μmol/l)	19.5 ± 20.7	15.9 ± 6.0	15.6 ± 4.4	*	*
C16SA (μmol/l)	0.54 ± 0.16	0.47 ± 0.25	0.37 ± 0.13	ns	***
C17SO (μmol/l)	8.1 ± 2.9	6.4 ± 2.1	6.5 ± 1.8	*	*
C17SA (μmol/l)	0.25 ± 0.05	0.22 ± 0.06	0.2 ± 0.04	ns	**
Phyto-SO (μmol/l)	0.3 ± 0.12	0.24 ± 0.09	0.23 ±0.07	ns	*
Sphingadiene (μmol/l)	29.8 ±7.8	25.6 ± 8.1	26.9 ± 6.3	ns	ns
Sphingosine (μmol/l)	82.3 ± 15.9	75.4 ± 16.6	74.7 ± 14.4	ns	ns
Sphinganine (μmol/l)	3.5 ± 1.0	3.4 ± 1.3	2.7 ± 0.7	ns	**
C19SO (μmol/l)	2.09 ± 0.82	1.82 ± 0.82	1.81 ± 0.71	ns	ns
C20SO (μmol/l)	0.2 ± 0.05	0.21 ± 0.08	0.19 ± 0.07	ns	ns
C20SA (μmol/l)	0.04 ± 0.02	0.04 ± 0.01	0.03 ± 0.01	ns	ns
1-deoxySO (μmol/l)	0.26 ± 0.15	0.34 ± 0.19	0.17 ± 0.04	*	*
1-deoxySA (μmol/l)	0.10 ± 0.07	0.12 ± 0.07	0.06 ± 0.02	ns	**

**CIAP**, chronic idiopathic axonal polyneuropathy; **DSPN**, (pre-)diabetic distal symmetrical polyneuropathy; **1-deoxy-SO**, 1-deoxy-sphingosine; **1-deoxy-SA**, 1-deoxy-sphinganine. Statistical analyses were performed with one-way ANOVA (using Tukey-Kramer post-test method to correct p-levels). The level of statistical significance is indicated as: not significant (ns): P > 0.05; significant:

*: P ≤ 0.05;

**: P ≤ 0.01;

***: P ≤ 0.001.

**Fig 1:**
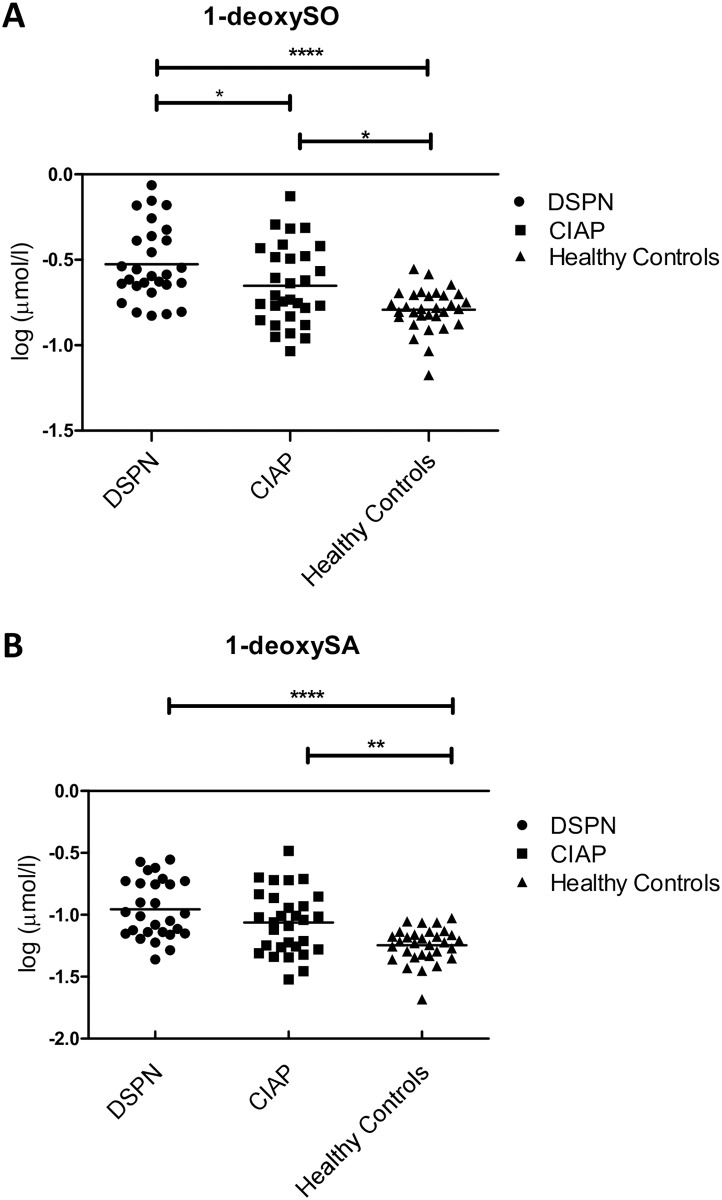
Distribution of 1-deoxySLs in plasma of CIAP-patients and controls. A significant elevation of 1-deoxySO (A) and 1-deoxySA (B) in plasma of CIAP- or DSPN-patients compared to healthy controls is shown. Not significant (ns): P>0.05; significant: *: P≤ 0.05; **: P≤ 0.01; ***: P≤ 0.001; ****: P≤ 0.0001.

The analysis of 13 different sphingoid bases in plasma showed a general elevation of long-chain-bases in CIAP-patients compared to the two control groups. Statistically significant was the increase of C_16_SO, C_16_SA, C_17_SO, C_17_SA, phyto-SO and sphinganine in CIAP-patients compared to healthy controls (Table 3). 1-deoxySO and 1-deoxySA were significantly elevated in both patient groups compared to healthy controls (Table 3, Fig 1). Almost equal numbers of patients with CIAP and DSPN (36.7%, n = 11 and 46.3%, n = 13, respectively), and only one healthy individual had abnormally elevated 1-deoxySO-levels. A 1-deoxySA increase was found in 30% (n = 9) of CIAP-patients, in 35.7% (n = 10) of DSPN-patients and in none of the healthy controls.

### Regression analyses of (para-)clinical features in CIAP and DSPN

In CIAP- and DSPN-patients, we performed regression analyses of metabolic parameters, 1-deoxySLs, clinical scores, sural nerve NCV and SNAP. No statistically significant association between laboratory values, such as TG- and cholesterol-levels or 1-deoxySLs and clinical and paraclinical parameters, indicating the severity of the polyneuropathy, could be established. Correlation between 1-deoxySLs to HDL-, LDL-cholesterol, TG and waist circumference showed the closest fit to TG-levels (vs.1-deoxySO R^2^ = 0.41; vs.1-deoxySA R^2^ = 0.39) and waist circumference (vs.1-deoxySO R^2^ = 0.19; vs.1-deoxySA R^2^ = 0.21), but did not reach statistical significance.

### Sural nerve biopsy studies in CIAP and DSPN (Table 4, Figs 2 and 3)

**Table 4:** Evaluation of sural nerve biopsies in patients with CIAP or DSPN.

Parameter	CIAP	DSPN	P
Number of biopsies	10	7	/
Number of fascicles	3.3 ± 1.9	4.3 ± 1.9	0.3 (ns)
Number of capillaries/fascicle	6.8 ± 3.9	6.2 ± 2.0	0.7 (ns)
Mean basal lamina thickness (μm)/capillary	3.5 ± 1.4	4.4 ± 0.9	0.2 (ns)
Mean number of endothelial cell nuclei/capillary	2.7 ± 0.4	2.9 ± 0.4	0.3 (ns)
Mean number of luminal endothelial cell nuclei/capillary	1.8 ± 0.3	2.0 ± 0.3	0.3 (ns)
Mean number of endothelial cell nuclei within basal lamina/capillary	0.8 ± 0.3	0.9 ± 0.3	0.6 (ns)
Number of CD68-pos. cells/fascicle	7.2 ± 4.6	5.1 ± 5.6	0.4 (ns)
Number of CD8-pos. cells/fascicle	1.9 ± 2.6	0.6 ± 0.4	0.4 (ns)
Number of LCA-pos. cells/fascicle	3.3 ± 2.3	1.2 ± 0.9	0.04 (*)

**CIAP**, chronic idiopathic axonal polyneuropathy: **DSPN**, (pre-)diabetic distal symmetrical polyneuropathy; **LCA**, leukocyte common antigen. Statistical analyses were performed using unpaired t-test. Statistical significance is indicated as: not significant (ns):

P > 0.05;

significant:

*: P ≤ 0.05.

**Fig 2:**
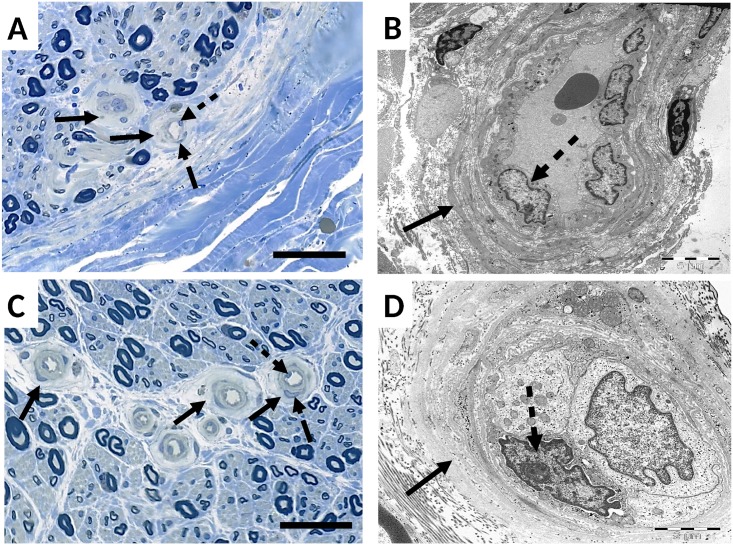
Light and electron microscopic images of sural nerve biopsies in CIAP (A, B) and DSPN (C, D). The basal lamina of small endoneurial vessels is prominently thickened (full arrows) in both conditions. Endothelial cell nuclei, located adjacent to the lumen (dotted arrows) or situated within the basal lamina (dashed arrows) are indicated. Magnification bar corresponds to 50μm in light microscope images (A, C) and to 5μm (B) and 2μm (D) in electron microscope pictures.

**Fig 3:**
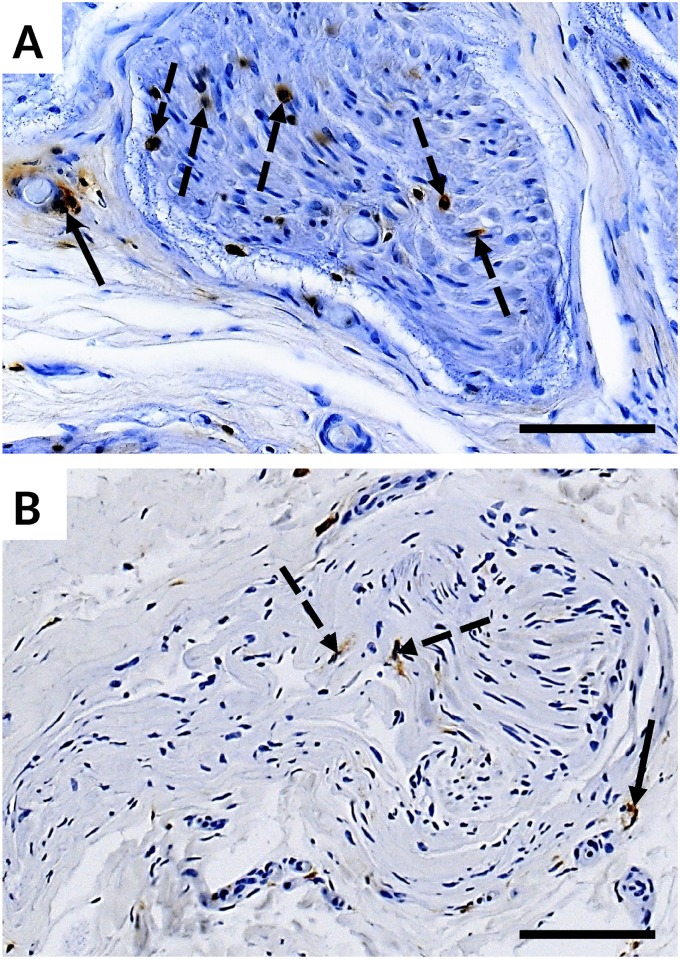
LCA immunohistological stains of sural nerve biopsies in CIAP (A) and DSPN (B). The number of endoneural (dotted arrows) and perivascular (full arrow) LCA-positive cells is significantly higher in biopsies of CIAP-patients compared to those in DSPN-patients. Magnification bar corresponds to 50 μm.

The numbers of small endoneurial vessels per fascicle in sural nerve biopsies did not differ significantly between CIAP- and DSPN-patients (P = 0.7), nor did the mean basal lamina thickness of the small endoneurial vessels and the mean number of endothelial cell nuclei per capillary (P = 0.2; P = 0.3). Differentiation between cell nuclei in basal lumen and within basal lamina also showed no significant difference between the two patient groups (P = 0.3; P = 0.6) (Table 4, Fig 2).

The number of CD68-positive and CD8-positive cells did not show significant differences. However, the number of LCA-positive cells was significantly increased in biopsies of patients with CIAP compared to DSPN (P = 0.04), with a mean number of LCA-positive cells per fascicle of 3.3 (±2.3) and 1.2 (±0.9), respectively (Table 4, Fig 3).

## Discussion

Our initial results indicated that the clinical and electrophysiological phenotype of CIAP and DSPN obtained by various objective neurological scores as well as NCV parameters are similar. Thus, we hypothesized that common factors such as the metabolic syndrome, elevated plasma 1-deoxySLs and microangiopathy might contribute to both conditions. Previous studies indicate a higher prevalence of metabolic risk factors such as IGT [6, 7], hypercholesterolemia [5], hypertriglyceridemia [4, 5], abdominal obesity and arterial hypertension [3] in CIAP-patients. We found a non-significant trend towards a higher occurrence of the metabolic syndrome in CIAP patients compared to healthy controls. There was, however, a significantly higher prevalence for hypercholesterolemia in CIAP compared to healthy individuals. In contrast, in DSPN patients, the metabolic syndrome was significantly more frequent than in healthy controls. In CIAP and DSPN patients, we found significantly raised 1-deoxySLs compared to healthy controls. Evaluation of sural nerve biopsies revealed microangiopathy in both CIAP and DSPN, and an additional inflammatory component in CIAP.

To examine whether the clinical phenotypes resemble each other, as it has been assumed [3], we attempt to objectively compare CIAP- to DSPN-patients. Due to the diagnostic criteria of CIAP a patient group with a slowly progressing, distal, predominantly sensory neuropathy [1] is pre-selected, similar as it is described for DSPN [24]. We found no differences in the ISS, MRC sum score or NCS results, emphasising the predominantly sensory character of both diseases and, if existent, the rather mild disabilities in motor function. Based on these phenotypic parallels, a common pathomechanistic pathway becomes conceivable. Significant differences, however, could be detected in pain severity, suggesting more small-fiber involvement in DSPN compared to CIAP [25, 26]. Calculations, furthermore, showed a trend towards significant differences in ONLS scores and mobility tests (P = 0.06; 0.053). This emphasises the small physical impairment and the slower progression of the disease in CIAP-patients [1], despite having a slightly longer duration of disease. Differences were mainly prominent when comparing ONLS, but not ISS scores. The ONLS score includes physical abilities and participation [18], whereas the ISS focuses on sensory impairment [16]. This again confirms that especially in CIAP the sensory impairment and not motor disabilities are the crucial disease characteristic [1].

CIAP-patients also showed a trend towards fewer signs of depression compared to DSPN-patients, which might be explainable by their better physical constitution, reflected in low ONLS scores and better mobility tests, as well as less severe pain [27].

Patients presenting IGT at 2h-oGTT were excluded from the CIAP-patient group in our study, eliminating one of five crucial risk factors for the diagnosis of a metabolic syndrome. DSPN-patients with DM2 have, by definition, a high prevalence for the metabolic syndrome since they always present IGT. This might explain, why we could not confirm that CIAP-patients have a significantly higher prevalence for the metabolic syndrome. In accordance with our results, other studies were only able to detect a higher prevalence of certain individual risk factors in CIAP, but not of the metabolic syndrome itself [4, 5]. Although 2h-oGTT and/or HbA_1c_ measurements in healthy controls might have provided an additional quantitative metabolic variable that could have been compared among all three groups, thus giving insight into the degree of long-term glucose control in CIAP patients and healthy controls, we did not include oGTT or HbA_1c_ data of healthy controls in our study.

In CIAP- and DSPN-patients 1-deoxySL plasma levels were significantly higher than in healthy controls. There was an almost equal percentage of CIAP- and DSPN-patients with abnormally high 1-deoxySLs, suggesting that in both CIAP and DSPN, the elevation is not caused by a few outliers, but is indeed a general feature in both disorders. These findings add on to a recent study, showing that neurotoxic 1-deoxySL-levels were exclusively elevated in DSPN-patients and not generally in all neuropathy patients [10]. The study concluded that 1-deoxySLs could crucially contribute to the disease mechanism of DSPN. Our results could implicate that 1-deoxySLs are a common pathomechanism for CIAP and DSPN, even though several reasons for the elevation in CIAP-patients have to be discussed. One approach that could explain the increase is the observation that 1-deoxySO and 1-deoxySA are not just raised in patients with diabetes mellitus, but also in patients with non-diabetic metabolic syndrome [28]. In our study, patients with CIAP showed a tendency towards the metabolic syndrome compared to healthy controls. Serine palmitoyltransferase (SPT) mainly metabolises L-serine, leading to the formation of sphinganine. SPT is also able to condensate L-alanine and palmitoyl-CoA creating 1-deoxySLs [11, 12]. Obesity is associated with an increased hepatic alanine uptake [29] which might result in an increased 1-deoxySL formation in the liver. Apart from the metabolic hypothesis, it cannot be ruled out that genetic variants, for example in the genes encoding the different subunits of the SPT, are associated with elevated 1-deoxySLs, in at least some of the cases, as it is for example known in patients with hereditary sensory and autonomic neuropathy type 1 (HSAN1) [30].

Our findings gain additional relevance since they might lead to new therapeutic options in CIAP-patients. Oral L-serine supplementation significantly lowered 1-deoxySL-levels in HSAN1 mouse models, as well as in a small pilot study with HSAN1-patients, and even improved neurological symptoms [31]. The same observation was made in a diabetic rat model [32].

A clear cut-off value defining microangiopathy does not exist. However, microangiopathy is a known neuropathological abnormality found in diabetic neuropathy [14] and was also demonstrated in biopsies of CIAP-patients [13]. In our study, both groups (CIAP and DSPN) showed equally increased thickness of the basal lamina of small endoneurial vessels, compatible with microangiopathy. Regression analysis of the basal lamina thickness to metabolic parameters, however, did not allow inference on the cause of enlargement. Our results stand in contrast to a recent publication by Samuelsson et al., which could not confirm a higher prevalence of microangiopathy in sural nerve biopsies of CIAP-patients [33]. The participants of our study (mean age 61.1 years) and the study of Teunissen et al. (mean age 63 years) [13] were older than in the study of Samuelson et al. (mean age 54.9 years). As suggested by Samuelsson and coworkers [33], this age difference might have contributed to the different findings, since correlations between basal lamina thickness and age have been demonstrated before [34]. An increased number of LCA-positive cells in sural nerve biopsies of CIAP-patients indicates in addition an inflammatory component as a possible mechanism contributing to the pathogenesis of CIAP. However, the number of LCA-positive cells was overall rather small (3.3 vs. 1.2 cells/fascicle).

A recent study suggested that autophagy might be another disease mechanism in CIAP [33], adding to the assumption of a multifactorial genesis in CIAP.

In conclusion, our study revealed profound clinical similarities between CIAP and DSPN, an elevation of neurotoxic 1-deoxySLs and nervous tissue microangiopathy in both disorders, which might be pathophysiological links. In addition, an inflammatory component could play a role in the pathogenesis of CIAP. Further research is needed to gain full understanding of the general pathophysiology in CIAP. It is conceivable that the disease has a multifactorial pathogenesis; however, 1-deoxySLs, basal lamina thickening of endoneurial vessels and possibly inflammation are new pieces of the puzzle in understanding nerve damage in CIAP.

## Supporting Information

S1 Table Demographic features and metabolic parameters in blood. (XLSX)

S2 Table Clinical scores. (XLSX)

S3 Table Nerve conduction studies. (XLSX)

S4 Table Plasma sphingolipids. (XLSX)

S5 Table Sural nerve biopsies. (XLSX)
